# Supplementary material for: Acute Kidney Injury Associated with Severe SARS-CoV-2 Infection: Risk Factors for Morbidity and Mortality and a Potential Benefit of Combined Therapy with Tocilizumab and Corticosteroids
Source: Biomedicines. 2023 Mar 10;11(3):845. doi: 10.3390/biomedicines11030845 (PMC10045336; doi:10.3390/biomedicines11030845)
Supplement: Supplementary file 1 [file biomedicines-11-00845-s001.zip › biomedicines-2230610-supplementary.pdf]

Supplemental table S1. missing values of Biomarkers

Biomarker	Valid values	Percent missing
D Dimer Day 1	112	55%
D Dimer Day 2	81	67%
CRP day 1	101	55%
CRP day 2	82	67%
Ferritin Day 1	119	56%
Ferritin day 2	105	58%
